# The Role of *AKT3* Copy Number Changes in Brain Abnormalities and Neurodevelopmental Disorders: Four New Cases and Literature Review

**DOI:** 10.3389/fgene.2019.00058

**Published:** 2019-02-22

**Authors:** Fátima Lopes, Fátima Torres, Gabriela Soares, Clara D. van Karnebeek, Cecília Martins, Diana Antunes, João Silva, Lauren Muttucomaroe, Luís Filipe Botelho, Susana Sousa, Paula Rendeiro, Purificação Tavares, Hilde Van Esch, Evica Rajcan-Separovic, Patrícia Maciel

**Affiliations:** ^1^School of Medicine, Life and Health Sciences Research Institute (ICVS), University of Minho, Braga, Portugal; ^2^ICVS/3B's - PT Government Associate Laboratory, Guimarães, Portugal; ^3^CGC Genetics, Porto, Portugal; ^4^Institute of Biomedical Sciences Abel Salazar (ICBAS), University of Porto, Porto, Portugal; ^5^Center for Medical Genetics Dr. Jacinto Magalhães, National Health Institute Dr. Ricardo Jorge, Porto, Portugal; ^6^Department of Pediatrics, Centre for Molecular Medicine, BC Children‘s Hospital, University of British Columbia, Vancouver, BC, Canada; ^7^Academic Medical Centre, Department of Pediatrics and Clinical Genetics, Amsterdam, Netherlands; ^8^Department of Pediatrics, Médio Ave Hospital Center, Vila Nova de Famalicão, Portugal; ^9^Medical Genetics Department, Hospital D. Estefânia, Centro Hospitalar Lisboa Central, Lisbon, Portugal; ^10^Department of Pediatrics, University of British Columbia, Vancouver, BC, Canada; ^11^Department of Neuroradiology, Hospital de Santo António, Porto Hospital Center, Porto, Portugal; ^12^Laboratories for Center for Human Genetics, University Hospitals Leuven, Leuven, Belgium; ^13^Department of Pathology, Children's Hospital of British Columbia, Vancouver, BC, Canada

**Keywords:** 1q43-q44 CNVs, *AKT3*, microcephaly, macrocephaly, *ZBTB18*, *SDCCAG8*, phenotypic expressivity

## Abstract

Microdeletions at 1q43-q44 have been described as resulting in a clinically recognizable phenotype of intellectual disability (ID), facial dysmorphisms and microcephaly (MIC). In contrast, the reciprocal microduplications of 1q43-q44 region have been less frequently reported and patients showed a variable phenotype, including macrocephaly. Reports of a large number of patients with copy number variations involving this region highlighted the *AKT3* gene as a likely key player in head size anomalies. We report four novel patients with copy number variations in the 1q43-q44 region: one with a larger deletion (3.7Mb), two with smaller deletions affecting *AKT3* and *SDCCAG8* genes (0.16 and 0.18Mb) and one with a quadruplication (1Mb) that affects the entire *AKT3* gene. All patients with deletions presented MIC without structural brain abnormalities, whereas the patient with quadruplication had macrocephaly, but his carrier father had normal head circumference. Our report also includes a comparison of phenotypes in cases with 1q43-q44 duplications to assist future genotype-phenotype correlations. Our observations implicate *AKT3* as a contributor to ID/development delay (DD) and head size but raise doubts about its straightforward impact on the latter aspect of the phenotype in patients with 1q43-q44 deletion/duplication syndrome.

## Introduction

The 1q43-q44 microdeletion syndrome is characterized by variable degrees of intellectual disability (ID), growth retardation, microcephaly (MIC), *corpus callosum* anomalies (CCAs), seizures (SZR), and abnormal facial features, such as round face with low-set ears, prominent forehead and flat nasal bridge, epicanthal folds and hypertelorism (De Vries et al., 2001; Ballif et al., 2012). The first report of pathogenic deletions at 1q43-q44 described a large microscopically observed deletion in a female patient with motor and mental impairment, MIC, SZR, and several dysmorphisms (Mankinen et al., 1976). With the development of microarray technology, many cases with submicroscopic deletions in this region were reported, with the consequent identification of the genes associated with the 1q43-q44 deletion syndrome (Boland et al., 2007; Hill et al., 2007; van Bon et al., 2008; Orellana et al., 2009; Caliebe et al., 2010; Lall et al., 2011; Nagamani et al., 2012; Wang et al., 2013). In Ballif et al. (2012) defined three potentially critical regions for MIC, CCAs, and SZR, proposing that MIC is associated with deletions of the *AKT3* (AKT serine/threonine kinase 3) gene (in 93% of the cases); CCAs with deletions affecting *ZNF238* (gene zinc finger and BTB domain containing 18, also called *ZBTB18*) (in 86% of the cases) and SZR with deletions affecting the *FAM36A* (also called *COX20*, cytochrome c oxidase assembly factor) and *HNRNPU* (heterogeneous nuclear ribonucleoprotein U) genes (in 87% of the patients) (Ballif et al., 2012). In the same year, Nagamani et al ruled out the implication of *AKT3* gene in CCAs, because patients 5 and 6 of their series, which have, respectively, an intragenic deletion of *AKT3* and a small deletion affecting *AKT3* and *SDCCAG8* (serologically defined colon cancer antigen 8) genes, did not have CCAs (Nagamani et al., 2012). The *HNRNPU* (heterogeneous nuclear ribonucleoprotein U) and the *FAM36A* (family with sequence similarity 36, also known as *COX20*—cytochrome c oxidase assembly factor) genes, were proposed as good candidates for the epilepsy and ID phenotype within the 1q43-q44 microdeletion syndrome (Thierry et al., 2012; Poot and Kas, 2013; Leduc et al., 2017). Even though the vast majority of the 1q43-q44 deletion cases described so far with MIC do carry genomic rearrangements that disrupt the *AKT3* gene, there are patients described in the literature with *AKT3* disruption that do not display MIC (Ballif et al., 2012). Conversely, there are also patients with 1q43-q44 deletion who display MIC even though they carry deletions that do not comprise the *AKT3* gene (Poot et al., 2008; van Bon et al., 2008; Ballif et al., 2012; Raun et al., 2017). In this perspective, the description of more patients with 1q43-q44 copy number variants (CNVs) may help to define more precise phenotype-genotype correlations. More recently, a deletion affecting exclusively the *AKT3* gene was described in a patient with MIC and ID and in his asymptomatic father, being the first report of a paternally inherited pure *AKT3* deletion of incomplete penetrance (Gai et al., 2015). In contrast with the deletions, there are only a few cases with pure gains in the region and detailed phenotypes. Copy number gains were described in patients with macrocephaly together with development delay, and also paired with speech and motor delay, hypotonia, and mild facial dysmorphisms (Wang et al., 2013; Chung et al., 2014).

The AKT3 protein belongs to the protein kinase B (PKB/Akt) family, involved in cell survival, proliferation and growth (Nakatani et al., 1999). In mice, both Akt1 and Akt3 play a role in determination of organ size. However, while Akt1 null mice have a decrease of all the organs, Akt3 null mice have a selective 20% decrease in brain size, Akt3 being the predominant Akt protein expressed in cortex and hippocampus. Unfortunately, the authors showed no data concerning the brain size in heterozygous animals, which would be relevant for the interpretation of the findings in humans (Easton et al., 2005). Akt3-null and heterozygous mice also have an impairment in angiogenesis, showing a dose-dependent reduction in vessel number (5-fold decrease in homozygous and 2.5-fold decrease in heterozygous), an aspect of the phenotype that was never evaluated in any of the reported patients (Corum et al., 2014).

We describe four patients with 1q43-q44 CNVs, detected by array comparative genomic hybridization (aCGH), and attempt to establish genotype-phenotype correlations, aiming to bring further insight into the role of *AKT3* in brain abnormalities and ID.

## Methods

### Ethical Procedures

Patients 1–3 were ascertained in the context of a larger study of neurodevelopmental disorders in Portugal, by the referring doctor. Clinical information was gathered in an anonymous database authorized by the Portuguese Data Protection Authority (CNPD). The study was approved by the ethics committee of Center for Medical Genetics Dr. Jacinto Magalhães, National Health Institute Dr. Ricardo Jorge. Written informed consent for sample collection, genetic studies and publication was obtained for all participants (signed by their parents). Informed consent for publication of photos was obtained from the parents for patients 1, 2, and 3 only.

### Molecular Karyotyping

Genomic DNA was extracted from peripheral blood using the Citogene® DNA isolation kit (Citomed, Portugal) for patient 1, QIAsymphony SP (QIAGEN GmbH, Germany) for patients 2 and 3 and DNeasy (QIAGEN GmbH, Germany) for patient 4. The aCGH hybridization and analysis was performed using: Patient 1—aCGH Agilent 180 K custom array (GEO GL15397, across-array methodology; Buffart et al., 2008; Krijgsman et al., 2013), Nexus Copy Number 5.0 software with FASST Segmentation algorithm for data analysis; Patient 2 and 3—Affymetrix CytoScan 750 K Platform (750.000 markers distributed throughout the genome, with a medium resolution of 8–20 Kb), Chromosome Analysis Suite (ChAS 3.0) software (Affymetrix); Patient 4—Affymetrix Cytoscan HD array.

### Quantitative PCR Confirmations

Primers for quantitative PCR (qPCR) were designed using Primer3Plus software (http://www.bioinformatics.nl/cgi-bin/primer3plus/primer3plus.cgi) and taking into account standard recommendations for qPCR primer development (Jovanovic et al., 2003).

For dosage quantification of *AKT3*, primers were designed for exons 8, 9, and 10 of the *AKT3* gene (ENSG00000117020). The reference genes used were *SDC4* (ENSG00000124145) and *ZNF80* (ENSG00000174255) localized in the 20q12-q13 and 3p12 regions, respectively (primers designed for all regions are listed Supplementary Table 1). All qPCR reactions were carried out in a 7,500-FAST Real Time PCR machine (Thermo Fisher Scientific, Waltham, MA, USA) using Power SYBR Green® (Thermo Fisher Scientific, Waltham, MA, USA). The specificity of each of the reactions was verified by the generation of a melting curve for each of the amplified fragments. The primer efficiency was calculated by the generation of a standard curve fitting the accepted normal efficiency percentage. Quantification was performed as described elsewhere (Hoebeeck et al., 2005). Ct values™ obtained for each test were analyzed in DataAssist™ software (Thermo Fisher Scientific, Waltham, MA, USA).

## Results

### Clinical Description and Molecular Findings

#### Patient 1 (DECIPHER #272238)

**Figure 1:**
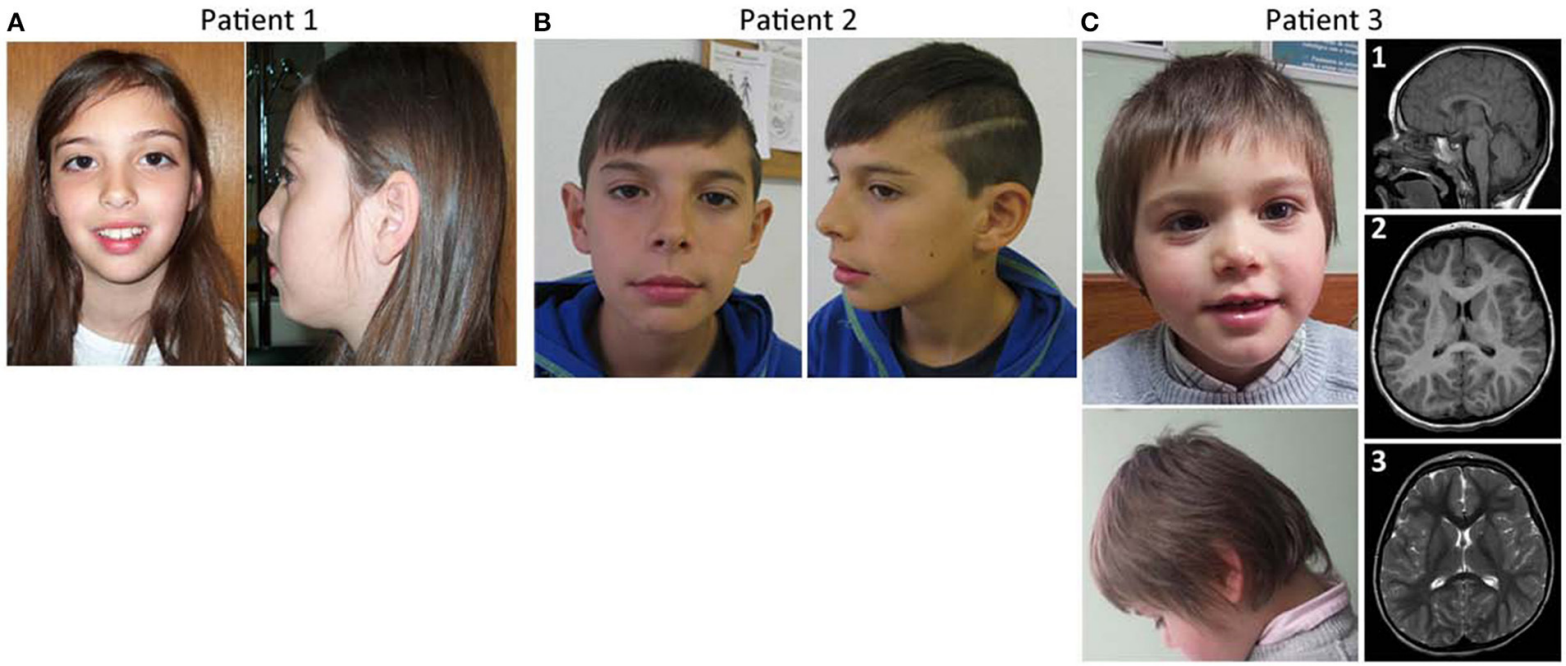
Facial features and brain imaging of the patients. **(A)** Patient 1 facial features, **(B)** patient 2 facial features, **(C)** Patient 3 facial features, and MRI brain imaging.

This girl was evaluated at 9 years of age for learning disabilities and MIC [occipitofrontal circumference (OFC) −2.5 SD]. Height and weight were at the 25th centile. She had a mildly sloping forehead and large upper central incisors (Figure 1A). Evaluation with the Wechsler Intelligence Scale for Children (third edition) (Wechsler, 1991) showed a full scale IQ of 63. Brain magnetic resonance imaging (MRI) was normal except for a discrete global atrophy. Pregnancy and delivery were uncomplicated at 35 weeks. OFC at birth was at the 3rd centile. Family history is unremarkable. Parents are young, healthy and non-consanguineous. The patient has a healthy younger brother. Congenital cytomegalovirus infection was excluded using PCR on the DNA obtained from the newborn metabolic disease screening Guthrie card. Informed consent was obtained from the child's parents for blood sampling and genetic analyses. Peripheral blood chromosome analysis demonstrated a normal 46,XX karyotype.

**Figure 2:**
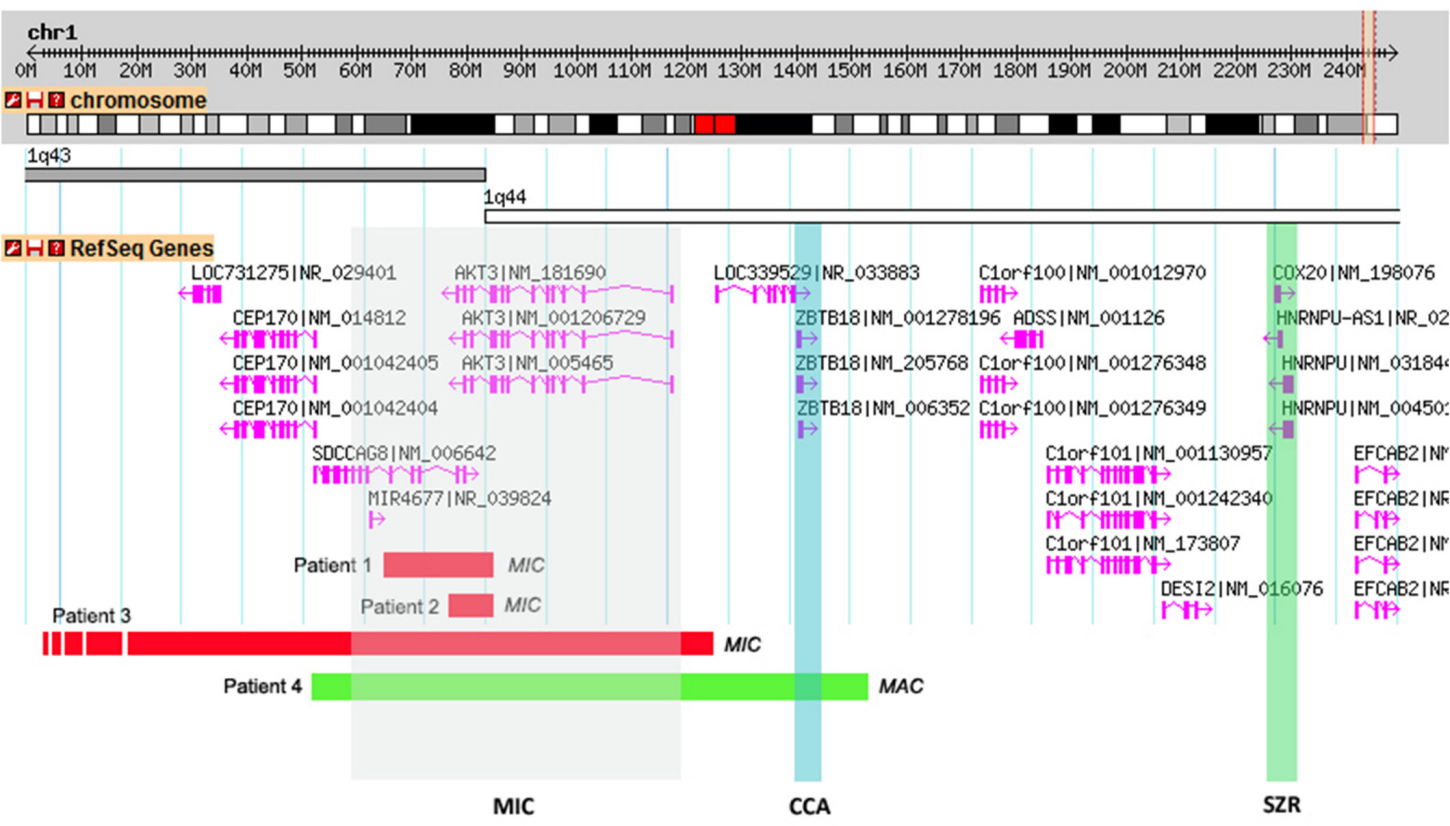
Schematic representations of the CNVs found in the patients and overlap with the critical regions proposed by Ballif et al. (2012). A 2.2Mb genomic portion encompassing cytobands 1q43-q44 is shown. RefSeq genes present within the genomic region are shown in pink and the transcriptional direction is shown by the arrows. Shaded in gray is the proposed critical region for microcephaly (MIC) (affecting the *AKT3* gene), in blue the critical region for *corpus callosum* anomalies (CCAs) (affecting the *ZNF238* gene) and in green the critical region for seizures (SZR) (affecting the *C1ORF199* gene). Individual red horizontal bars represent deletions and the green bar a quadruplication. In each CNV the corresponding patient is indicated.

Subsequent array CGH revealed a 0.18 Mb *de novo* deletion at chromosome region 1q43-q44 (chr1:243,552,007–243,738,675) containing the *AKT3* and *SDCCAG8* genes (Figure 2). The 1q43-q44 deletion was confirmed by qPCR for the *AKT3* gene (exons 7, 8, 9, and 10). This analysis showed the deletion breakpoint to be located between exons 8 and 9. Analysis of the same fragments in both parents showed that the deletion occurred *de novo*. Sanger sequencing of the *AKT3* coding region revealed no variants.

#### Patient 2 (DECIPHER # 367117)

This boy was referred for evaluation of MIC and learning difficulties, associated with global DD in the past. Development evaluation (Griffiths) performed in 2011 reported all developmental areas within the low inferior range. He has an OFC of 48 cm (*P* < 1, −3.9 SD) and presents slightly dysmorphic features, including long philtrum and thin upper lip with cupid's bow (Figure 1B). Brain MRI performed at age 4 years was normal. Presently, 12 years old, he attends a school with an adapted curriculum, maintaining some learning difficulties. He was treated with risperidone for aggressive behavior in the past, but treatment has been discontinued. No seizures or other behavior anomalies were reported. The patient has a healthy older sister who was not genetically tested. Learning difficulties were also reported in the paternal side of the family: the father has MIC (OFC of 52.34 cm, P3), presents a rather long face, and has mild learning difficulties; one paternal uncle can't read or write and has a son with learning difficulties; a sister of the great-great-grandmother was always assistance-dependent due to a supposed ID and MIC. Apart from the father, none of the affected family members was genetically tested.

Array CGH revealed a 0.16 Mb deletion (chr1:243,592,147–243,749,968) affecting both the *AKT3* and *SDCCAG8* genes (Figure 2). The presence of the deletion was confirmed by qPCR for the *AKT3* gene (exon 10), which also revealed paternal inheritance.

#### Patient 3 (Decipher # 367116)

This boy was referred for evaluation around 3–4 months of age for evident MIC. At 3 years of age he presented weight and height within the normal range, but his OFC has been in P1 (−2.6 SD) since he was 8 months old. The patient had a hyperkinetic behavior and global DD, the language delay being the most striking. At the age of 5 years he was undertaking speech therapy. He didn't know colors or numbers, was described by parents as “clumsy” and by the teacher as aggressive. The patient has type B bilateral tympanogram. MRI evaluation of the brain at 3 years and 8 months old showed a cerebral volume in accordance with a decreased cephalic perimeter, without enlarged cerebrospinal fluid spaces [Figure 1C, (1–3)]. The cerebral hemispheres appeared otherwise unremarkable without noticeable malformations of cortical development, no signs of hypoxic-ischemic or infectious lesions. The *corpus callosum* was completely formed and displayed a normal thickness. No other abnormalities were seen. There was no evidence of significant skull abnormalities, other than the identified smaller dimensions and a slight left positional plagiocephaly; the electroencephalogram (EEG) was also normal. Concerning family history, he has a maternal uncle with epilepsy.

Array CGH revealed a 3.7 Mb deletion (chr1:240,366,425–244,111,022) (Figure 2), that proved to be *de novo* after qPCR confirmation using primers for the *AKT3* gene (exon 10).

#### Patient 4 (DECIPHER # 367118)

This boy was born at term, after an uneventful pregnancy and delivery, with poor Apgar scores, absence of gag reflex. Initially he presented hypotonia and apnea, having developed seizures and dystonia at a later age (all symptoms appeared before 5 years of age). He had a suspected IQ below 70 (although no formal evaluation was performed). He also presents macrocephaly (with OFC of 55.5 cm at 7 years old, corresponding to the 99th centile, +2.53 SD) and white matter lesions of brain including thalamic lesions. aCGH revealed a 1 Mb paternal quadruplication (chr1:243,415,063–244,478,355) (Figure 2) affecting the *CEP170, AKT3, SDCCAG8*, and *ZBTB18* genes; the father is phenotypically normal, even though he is a carrier of this genetic variant. Meanwhile, patient 4 also underwent trio whole exome sequencing analysis, which retrieved no diagnosis.

## Discussion

In this study, the findings in patients 1, 2, and 3 would support the conclusion that haploinsufficiency of *AKT3* gene is indeed associated with microcephaly. Comparing patient 6 described by Nagamani et al. (2012) with patients 1 and 2 of this report, MIC in our patients may be explained by the presence of a deletion that affects a bigger portion of *AKT3* (it affects the last 4 exons of *AKT3*), while in the patient presented by Nagamani the deletion only affects the last 2 exons of the gene. Patient 3 in the current study fits quite well in the established 1q43-q44 microdeletion syndrome regarding the phenotype and size of the deletion. This patient, despite having a larger deletion that includes not only the *AKT3* gene but also the *CEP170* gene, has evident MIC but an apparently normal *corpus callosum* (Figure 1C).

The association of *AKT3* copy number gains with the mirror phenotype (macrocephaly) has also been reported in literature (Wang et al., 2013; Chung et al., 2014; Conti et al., 2015; Hemming et al., 2016). *AKT3* is one of the genes in which this type of mutations was found in patients with severe overgrowth syndromes (Tatton-Brown and Weksberg, 2013). Patient 4 in our series does present macrocephaly, supporting this model. However, his father becomes the first reported case of a quadruplication affecting *AKT3* in an asymptomatic individual.

In 2008, van Bon et al. described a pair of sisters with ID and MIC who inherited an *AKT3*-affecting deletion from their healthy mother (patient 11 and 12 from their series; the mother had normal IQ and normal OFC). In Ballif et al. (2012) also described a patient series in which three patients had inherited 1q43-q44 deletions (patient 10 which has MIC, 21 which has no information regarding OFC and 22 which doesn't have MIC). However, only patient 10 carried a deletion that affected *AKT3* and it was maternally inherited. Reports of cases carrying 1q43-q44 CNVs with different clinical outcomes can also be found in the DECIPHER database (Firth et al., 2009). There are three DECIPHER cases with *AKT3*-affecting deletions inherited from parents (#317423, #252432, and #277172, the two latter ones being reportedly healthy progenitors). However, it is important to highlight the difficulties in interpreting this data given the often incomplete and/or imperfect annotation of DECIPHER entrances, particularly regarding clinical description and follow up studies.

A possible contributing factor for the incomplete penetrance and clinical variability associated with *AKT3* genetic variants may be somatic mosaicism. This could lead to the heterogeneous distribution of AKT3 and/or modifying gene variants at the somatic level. In fact, somatic mutations activating the mTOR pathway have been shown to cause a continuum of cortical dysplasias; ultra-deep sequencing on DNA extracted from surgically resected brain, blood, and/or buccal samples from patients with several (mostly focal or asymmetric) cortical malformations led to the identification of somatic activating mutations in several mTOR genes, including *AKT3* (Lee et al., 2012; Poduri et al., 2012; Alcantara et al., 2017; D'Gama et al., 2017). Moreover, D'Gama et al. have proposed a “two-hit” model in a patient with both germline and somatic *TSC2* gene mutations (D'Gama et al., 2017). The presence of “second-hit” mutations, undetectable by targeted sequencing or present at a level below the detection limit of the techniques commonly used, could therefore contribute to the variability of the phenotypes presented. This may be paricularly relevant in a situation of increased gene dosage, which *per se* could have a similar but more subtle impact than *AKT3* somatic gain of function mutations.

A comparison between the “core” 1q43-q44 deletions and duplications phenotype and the four reported cases is made in Table 1. Of notice, the number of cases reported with duplications affecting the *AKT3* is quite reduced, making it difficult to establish a core symptomatology.

**Table 1:** Comparison of the clinical features of the patients in the current series with patients with *AKT3* deletions described in the literature.

**Clinical feature**		**Core phenotype**	**Patient 1**	**Patient 2**	**Patient 3**	Core phenotype^*^	**Patient 4**
CNV		CN Loss	Deletion	Deletion	Deletion	CN Gain (pure)	Quadruplication
Clinical Overview	Gender	NR	♀	♂	♂	NR	♂
	Consanguinity	NR	No	No	No	NR	No
	Birth	NR	35 w (uneventful)	41 w	40 w	NR	To term
	Measurements at birth (heigh/weigth/OFC)	NR	NA	49cm (P7)/3240g(P10)/33cm(P2)	47cm(P1)/2710g(P2)/32.5cm(P1)	NR	NA
	Age at observation	NR	9y	12y	3y (Evident MIC)	NR	Before 5y
	ID	Moderate to severe	Mild (IQ = 63)	Mild (IQ NA)	Mild (IQ = 61)	Moderate	Mild
	Weigth (centile) at observation	NR	P25	*P* < 5	Within normal range for age^¥^	NR	P50
	Heigth (centile) at observation	Short stature	P25	P3	Within normal range for age^¥^	NR	P20
Cranio-facial abnormalities	Head/ OFC (centile)	MIC	MIC (*P* < 3); −2,5SD	MIC (*P* < 5)	MIC (*P* < 3)	MAC	MAC
	Structural brain abnormalities	Corpus callosum abnormalities (CCA) (agenesis/hypogenesis)	No	No	No	No	No
	Facial dysmorphisms	Round face, prominent forehead, flat nasal bridge, hypertelorism, epicanthal folds, malformed and low-set ears, hand and foot anomalies,	Mildly sploping forehead; large upper incisors	No major dysmorphisms	Upward palpebral fissures; retrognatia; poor hearing (conduction); dental caries	Prominent forehead, hypertelorism, wide nasal bridge, horizontal palpebral fissures, low set protruding ears.	NA
Others	Behavior	No alterations	No alterations	Aggressivity	Aggressivity; Auto-mutilation hyperactivity	No alterations	No alterations
	Seizures	Yes	No	No	No	No	No
	Hypotonia	Yes	No	No	No	Yes	No
	Genitalia	No alteration pattern	Normal	NA	Normal	No alteration pattern	Normal
	Heart	Minor heart conditions (usually resolve naturally)	NA	NA	Never evaluated	No	No
Molecular findings	Inheritance	–	*de novo*	Paternal	*de novo*	–	Paternal
	Confirmation	–	qPCR	qPCR	qPCR	–	NP
	Start (hg19)	–	243,552,007	243,592,147	240,366,425	–	243,415,063
	End (hg19)	–	243,738,675	243,749,968	244,111,022	-	244,478,355
	Size (Mb)	–	0.18	0.16	3.7	–	1
	Genes affected	–	*AKT3, SDCCAG8*	*AKT3, SDCCAG8*	*FMN2, GREM2, RGS7, FH, KMO, OPN3, CHML, WDR64, EXO1, MAP1LC3C, PLD5, LOC731275, CEP170, SDCCAG8, AKT3*	–	*CEP170, AKT3, SDCCAG8, ZNF238*

* *Core phenotype of patients with 1q43–q44 duplications should be interpreted with care as there are very few cases reported*.

¥ *Specific measure not available; CNV, copy number variants; NA, not available; OFC, occipitofrontal circumference; y, years; m, months; MIC, microcephaly; ID, intellectual disability; DD, developmental delay; NR, not representative; qPCR, quantitative PCR; ND, not determined; NP, not performed*.

The lack of objective phenotypic measures among 1q43-q44 CNVs reports is an important limitation, however, to the adequate establishment of genotype-phenotype correlations. This was recently exposed by Raun et al who, by using more rigorous measures of head size deviation, showed that *AKT3* deletion is associated with more severe forms of MIC, while deletions in 1q43-q44 not affecting *AKT3* resulted in less severe MIC (probably because, as suspected before, *AKT3* is unlikely to be the only gene modulating head size at the 1q43-q44 region) (Raun et al., 2017). However, this might not be the case for all the cases since our patients don't seem to follow this pattern. Patient 3 has a MIC with −2.6 SD below the mean even though he has a deletion altering several gene besides *AKT3*, whereas patient 1, who only has the *AKT3* and *SDCCAG8* genes deleted, has a very similar MIC (−2.5 SD) to that of patient 3. Patient 2 is the case with the more severe MIC, with a SD of −3.9 even though he is the one with the smallest deletion of our series. *AKT3* partial or pure deletions may thus be subject to incomplete penetrance and/or differential expressivity driven by different genetic and epigenetic backgrounds of the individuals (the resulting phenotype not necessarily related to the deletion size). Although never reported in the 1q43-q44 region nor affecting *AKT3* specifically (the *AKT3* gene is not listed in the Geneimprint database on April 2018) (Geneimprint : Genes), imprinting alterations constitute another mechanism of differential growth (dy)sregulation which could be of relevance in these patients (reviewed in Choufani et al., 2013; Geneimprint, 2018).

Recently, single nucleotide variations in *ZBTB18* were identified in two patients, one with developmental and speech delay, MIC and dysmorphic features and the other with severe ID, breathing disturbances and MIC without structural anomalies (de Munnik et al., 2014; Lopes et al., 2016). This shows that *ZBTB18* mutation is sufficient to cause MIC, contradicting the exclusive contribution of *AKT3*. Although, this gene is not directly involved in the three patients with deletions here described, we cannot exclude that the deletions occurring in patient 1, 2, and 3 don't have an effect in *ZBTB18* expression in the nervous system.

The minimal overlapping region of all the patients with 1q43-q44 CNVs described in the current study encompasses only one additional gene to *AKT3*: the *serological defined colon cancer antigen 8* (*SDCCAG8*). This gene encodes for a protein thought to be a stable centrosomal component with a structural role in the centrosomal architecture or the microtubule-organizing activities of the centrosome matrix (Kenedy et al., 2003). Mutations in *SDCCAG8* were described in patients with nephronophthisis-related ciliopathies; even though the clinical features of those patients include ID, a feature that is present in our patient, the alterations causing disease in those cases are associated with a recessive model of inheritance (Otto et al., 2010). Recessive variants in *SDCCAG8* gene were also associated with Bardet-Biedl syndrome and with an increased risk for schizophrenia (Schaefer et al., 2011; Hamshere et al., 2013). Additionally, *SDCCAG8* has been described to play a role in neuronal polarization and migration in the developing mouse cortex (Insolera et al., 2014), which would be consistent with the described genetic effects in humans. However, and given the presence of *SDCCAG8* deletions and quadruplication in heterozygosity in our patients, it most likely is not contributing to the MIC phenotype.

In summary, we describe four patients with 1q43-q44 CNVs, three of which with outcomes that are quite consistent with those of the “core” 1q43-q44 deletions affecting the *AKT3* gene, whereas the last (patient 4), in combination with other previously reported cases, highlights the not so straightforward and isolated implication of *AKT3* CNVs in human OFC determination. Despite its known biological function and the strong evidence that *AKT3* is a key gene for MIC in patients with 1q43-q44 deletions, other factors must play a role in the arising of the phenotype, resulting in incomplete penetrance and variable expressivity, perhaps the consequent of different genetic or epigenetic backgrounds of the individuals. This variability has important implications in the clinical practice in the context of the genetic counseling. The implication of *AKT3* in head size appears to be clear for the vast majority of the cases, even though not absolute. For this reason, the reporting of more patients with 1q43-q44 CNVs, their clinical and genetic features and their variable phenotypic expressivity is important and should be continued.

## Author Contributions

FL, FT, SS, PM, and PR performed the molecular studies and analyzed the molecular data. GS, CvK, CM, DA, JS, LM, and LB collected and analyzed clinical data. FL, FT, ER-S, HV, and PM drafted the paper. PT, ER-S, and PM obtained funding for this study. The study was performed under the direction of PM. All authors have agreed with and approved the final version.

### Conflict of Interest Statement

FT, PR, and PT were employed by the company CGC Genetics. The remaining authors declare that the research was conducted in the absence of any commercial or financial relationships that could be construed as a potential conflict of interest.
