# Supplementary material for: The Role of AKT3 Copy Number Changes in Brain Abnormalities and Neurodevelopmental Disorders: Four New Cases and Literature Review
Source: Front Genet. 2019 Feb 22;10:58. doi: 10.3389/fgene.2019.00058 (PMC6395382; doi:10.3389/fgene.2019.00058)
Supplement: Supplementary file 1 [file Table_1.DOCX]

Supplementary material to the article:

**The role of *AKT3* copy number changes in brain abnormalities and neurodevelopmental disorders: four new cases and literature review**

Fátima Lopes^1,2^, Fátima Torres^3,4^, Gabriela Soares^5^, Clara D. van Karnebeek^6,7^, Cecília Martins^8^, Diana Antunes^9^, João Silva^5^, Lauren Muttucomaroe^10^, Luís Filipe Botelho^11^, Susana Sousa^1,2^, Paula Rendeiro^3^, Purificação Tavares^3^, Hilde Van Esch^12^, Evica Rajcan-Separovic^13^, Patrícia Maciel^1,2*^

^1^Life and Health Sciences Research Institute (ICVS), School of Medicine, University of Minho, Braga, Portugal

^2^ICVS/3B’s - PT Government Associate Laboratory, Braga/Guimarães, Portugal

^3^CGC Genetics, Porto, Portugal

^4^Institute of Biomedical Sciences Abel Salazar (ICBAS), University of Porto, Porto, Portugal;

^5^Center for Medical Genetics Dr. Jacinto Magalhães, National Health Institute Dr. Ricardo Jorge, Praça Pedro Nunes, Porto, Portugal

^6^Department of Pediatrics, BC Children‘s Hospital & Centre for Molecular Medicine, University of British Columbia, Vancouver, Canada

^7^Department of Pediatrics, Médio Ave Hospital Center, Vila Nova de Famalicão, Portugal

^8^Medical Genetics Department, Hospital D. Estefânia, Centro Hospitalar Lisboa Central, Lisboa, Portugal

^9^Department of Neuroradiology, Hospital de Santo António, Porto Hospital Center, Porto, Portugal

^10^Laboratories for Center for Human Genetics, University Hospitals Leuven, Leuven, Belgium.

Correspondence to:

Patricia Maciel, PhD

Life and Health Sciences Research Institute (ICVS) - School of Medicine, University of Minho, Campus de Gualtar, 4710-057 Braga, Portugal

Phone: +351 253604824

Email: [pmaciel@ecsaude.uminho.pt](mailto:pmaciel@ecsaude.uminho.pt)

**Supplementary Table S1 – Primers used for quantitative PCR confirmation.**

| **Chromosome** | **Gene** | **Reference sequence** | **Primer location** | **Primer Forward 5'🡪3'** | **Primer Reverse 5'🡪3'** | **Amplicon size (bp)** |
| --- | --- | --- | --- | --- | --- | --- |
| Chr 1 | *AKT3* | ENSG00000117020 | Exon7 | TCTGGGCTTAACCTCTTCCA | TGTTAAAAAGGGATGTCTAGTGTTC | 162bp |
| Chr 1 | *AKT3* | ENSG00000117020 | Exon8 | CCTTGAAATATTCCTTCCAGACA | CCATGCAAATACTGGATTTACTTCT | 101bp |
| Chr 1 | *AKT3* | ENSG00000117020 | Exon9 | AGAGAGCGGGTGTTCTCTGA | CCTTGAGATCACGGTACACAA | 106bp |
| Chr 1 | *AKT3* | ENSG00000117020 | Exon10 | CAGTTGGAGAATCTAATGCTGGA | AATGGAACCGAAGCCTACCT | 150bp |
| Chr 3 | *ZNF80* | ENSG00000174255 | Exon1 | GCTACCGCCAGATTCACACT | AATCTTCATGTGCCGGGTTA | 182bp |
| Chr 20 | SDC4 | ENSG00000124145 | Exon4 | ACCGAACCCAAGAAACTAGA | GTGCTGGACATTGACACCT | 101bp |
